# Psychosocial correlates of self-reported HIV among youth in the slums of Kampala

**DOI:** 10.1186/s12889-019-7480-z

**Published:** 2019-08-27

**Authors:** Monica H. Swahn, Rachel Culbreth, Laura F. Salazar, Nazarius M. Tumwesigye, Rogers Kasirye

**Affiliations:** 1 Department of Population Health Sciences, School of Public Health, Georgia State University, P.O. Box 3984, Atlanta, GA 30302-3984 USA; 2 Department of Respiratory Therapy, Byrdine F. Lewis College of Nursing and Health Professions, Georgia State University, P.O. Box 3995, Atlanta, GA 30302-3995 USA; 3 Department of Epidemology and Biostatistics, School of Public Health, Makerere University, PO Box 7062, Kampala, Uganda; 4 Uganda Youth Developmental Link, P.O. Box 12659, Kampala, Uganda

**Keywords:** AIDS/HIV, Alcohol, Social epidemiology, Child health

## Abstract

**Background:**

Human immunodeficiency virus (HIV) rates are high in Uganda (6.7%), and rates are especially high among at-risk groups such as youth living in the slums of Kampala, Uganda. The objective of this study was to assess the psychosocial correlates, particularly alcohol use, associated with HIV among youth living in the slums of Kampala, Uganda.

**Methods:**

Analyses are based on cross-sectional survey data collected in Spring of 2014. Participants comprised a convenience sample (*N* = 1134) of urban service-seeking youth living on the streets or in the slums, 12–18 years of age who were participating in a Uganda Youth Development Link drop-in center (56.1% female and 43.9% male). Chi-Square Tests were used to determine differences in the proportions of alcohol use patterns between self-reported HIV-positive and HIV-negative youth. Bivariate and multivariable logistic regression were conducted to determine the associated risk factors with self-reported HIV. Institutional Review Board approvals were obtained from the Georgia State University and the Uganda National Council for Science and Technology.

**Results:**

Among the total sample of youth (*N* = 1103), 10.5% (*n* = 116) reported being HIV-positive. There were statistically significant differences between HIV-positive and HIV-negative youth on ever living on the streets (*χ*^2^ =10.14, df = 1, *p* = 0.002), past 12-month alcohol use (*χ*^2^ =16.38, df = 1, *p* < .0001), ever having sexual intercourse (*χ*^2^ =14.52, df = 1, *p* = 0.0001), ever engaging in sex work (*χ*^2^ =13.19, df = 1, *p* = 0.0003), inconsistent condom use in the past 3 months (*χ*^2^ =5.03, df = 1, *p* = 0.03), and ever being raped (*χ*^2^ =15.29, df = 1, *p* < 0.0001). A higher percentage of HIV-positive youth were classified as problem drinkers, defined by the CAGE scores (21.6% vs. 13.9%, respectively). In the multivariable analysis, previously being raped (OR: 1.70; 95% CI: 1.02, 2.83) and alcohol use without problem drinking (OR: 2.14; 95% CI: 1.24, 3.69) was associated with HIV.

**Conclusion:**

Youth living in the slums of Kampala, Uganda have a high prevalence of HIV. These youth are in dire need of interventions which address both alcohol use behaviors and sexual risk behaviors to reduce further complications of their existing health conditions, including HIV.

## Background

Sub-Saharan Africa has an estimated 24.7 million people who are living with human immunodeficiency virus (HIV), accounting for nearly 71% of the global burden [1]. Uganda has an estimated 6.5% HIV prevalence [2] and is one of two countries in Africa where HIV rates are increasing instead of decreasing [1]. Certain groups are disproportionately burdened by HIV within Uganda and include young women, commercial sex workers, men who have sex with men, and youth living in the slums of Kampala [1]. For example, HIV prevalence among sexually active youth living in the slums of Kampala (13.9%) is higher than the national prevalence [3], and HIV infection may be exacerbated in this population by a lack of adequate infrastructure, food scarcity, and limited or no parental oversight [4–7]. In addition, complex, synergistic factors, such as gender-based violence [8–12], alcohol use [13–15], and engagement in commercial sex work also play a significant role [3, 16–19], although alcohol use is at the center of these factors.

Alcohol use is known to impair judgment leading to high-risk sexual behaviors, including unprotected sex and multiple sex partners [14, 20, 21]. Alcohol use is highly prevalent among youth living in the slums of Kampala [3–5]. Nearly 1/3 of youth living in the slums have reported drunkenness [5], and research has shown that *heav*y alcohol consumption is more strongly linked to HIV acquisition as compared to *frequency* of alcohol consumption [14]. Gender differences also exist in the relationship between alcohol use and HIV. For males, heavy alcohol consumption is strongly linked to engaging in high-risk sexual behaviors, whereas women’s risks for HIV acquisition are mostly dependent on their partner’s heavy alcohol consumption [14].

Alcohol use is also common in many gender-based violence episodes [8] and commercial sex work engagement with clients, both of which are risk factors for HIV acquisition [3, 18]. The mechanisms linking gender-based violence and HIV include forced unprotected sex and psychological trauma negatively impacting condom use negotiation [8]. Additionally, alcohol use further increases the incidence of these behaviors [8]. Commercial sex work engagement is strongly linked to HIV acquisition through unprotected sex and forced sexual encounters [17, 22–25]. Again, alcohol use exacerbates the link between commercial sex work and HIV acquisition [18]. In a study in Malawi, sex workers who consumed alcohol were four times more likely to not use a condom compared to sex workers who did not consume alcohol [26]. Alcohol consumption is also common among youth living in the slums of Kampala who are engaging in sex work [3]. In fact, nearly 90% of youth engaging in sex work have drank alcohol, and approximately 40% of the youth engaged in sex work were paid in alcohol [3].

Both HIV and alcohol use are highly prevalent among youth living in the slums of Kampala [3, 5]. No study to our knowledge has documented the overall burden of HIV and HIV-related risk behaviors among youth living in the slums of Kampala. This population is particularly vulnerable due to environmental conditions, such as extreme poverty, limited infrastructure, lack of healthcare access, in addition to many psychosocial conditions, including alcohol use, commercial sex work, parental physical abuse and sexual abuse [4–7]. Because of these adversities and unique vulnerabilities of this population, predictors of HIV may be different in this population compared to other populations. The purpose of this study is to determine the demographic characteristics, high-risk sexual behaviors, alcohol-related behaviors, and gender differences associated with self-reported HIV.

## Methods

### Setting

The current study is based on the “Kampala Youth Survey 2014”, a cross-sectional survey conducted in the spring of 2014 to quantify and examine high-risk behaviors, with a focus on alcohol use, sexual behaviors and HIV. The study population consisted of urban youth, between 12 and 18 years of age, living in the slums or on the streets of Kampala, Uganda, who were participating in a Uganda Youth Development Link (UYDEL) drop-in center for disadvantaged street and slum youth [27]. Study participants were recruited at six drop-in centers and the neighborhoods surrounding the UYDEL drop-in centers primarily through word-of-mouth.

### Data collection

During the data collection period, 1628 youth were approached to participate in the study. Of these youth, 131 declined yielding a participation rate of 92% and a total of 1497 interviews were conducted. Three hundred and twenty (320) interviews were lost due to technical issues with the offline server, yielding a final analytic sample of 1134 (44% boys, 56% girls).

Interviewers consisted of social workers and peer educators from UYDEL who have previous experience working with the youth. Verbal consent was obtained from participants. According to conditions under the Uganda National Council for Science and Technology, youth who “cater for their own livelihood” are considered emancipated in Uganda and are able to provide their own consent for participation. Inclusion criteria included youth ages 12–18. Institutional Review Board approvals were obtained from Georgia State University and the Uganda National Council for Science and Technology to conduct this study in Kampala (SS3338).

The Kampala Youth Survey 2014 consisted of measures on alcohol use and alcohol marketing, violence exposures, sexual risk behaviors and HIV, and mental health. Study questions were obtained from previously validated instruments used in the United States and globally, including: Global School-based Student Health Survey (GSHS) [28], Kampala Youth Survey 2011 [3–7], the Monitoring Alcohol Marketing Practices in Africa 2012 Questionnaire, the Alcohol Use Disorders Identification Test (AUDIT) Questionnaire [29], the CAGE Questionnaire [30], a safer sex messages intervention questionnaire [31], the Acquired Immunodeficiency Syndrome (AIDS) Indicator Survey [32], and the Demographic Health Survey [33].

### Measures

The presence of HIV was self-reported in this study. Respondents were classified as HIV-positive if they answered, “Yes” to “Have you been told by a doctor/nurse or HIV counselor that you have HIV?” Respondents were classified as HIV-negative if they answered, “No.” Problem drinkers and non-problem drinkers were classified using the CAGE questionnaire [30]. The CAGE questionnaire consisted of four questions which comprise the acronym CAGE (“Have you ever felt you should **C**ut down on your drinking?”; “Have people **A**nnoyed you by criticizing your drinking?”; “Have you ever felt bad or **G**uilty about your drinking?”; and “Have you ever had a drink first thing in the morning to steady your nerves or to get rid of a hangover (**E**ye opener)?”) [30]. Items are scored 0 or 1 and a higher score indicates a more severe alcohol problem. Scores of 2 or more are considered “clinically significant,” and therefore, classified as problem drinkers [30]. Other questions regarding alcohol use were assessed, including age at first drunkenness, age at first alcohol beverage, frequency of alcohol consumption, and questions about alcohol-related risky behaviors. Other psychosocial measures included ever living on the streets, commercial sex work, condom use at last sex, and ever being raped.

### Data analysis

Descriptive statistics for demographic characteristics, high-risk behaviors, and alcohol-related behaviors were computed among self-reported HIV-positive and self-reported HIV-negative youth. Thirty-one (*n* = 31) youth were excluded from the analysis because they were missing on the self-reported HIV measure. Descriptive statistics were also computed for demographics and risk-behaviors between female and male HIV-positive youth. Chi-square tests were performed to determine statistically significant differences (*p* < 0.05), and Fisher Exact Tests were used when expected cell sizes were less than 5. Finally, a multivariable analysis was conducted to determine the statistically significant factors related to self-reported HIV. Odds ratios and 95% confidence intervals are reported from bivariate and multivariable logistic regression results. All analyses were conducted in SAS 9.4 (SAS Institute, Cary, NC).

## Results

Among the total sample of youth (*N* = 1103), 10.5% (*n* = 116) reported being HIV-positive (Table 1). The median age among self-reported HIV-positive and self-reported HIV-negative youth was 17 (IQR = 3) years of age. There were no statistically significant differences in proportions between self-reported HIV-positive and self-reported HIV-negative youth on demographic characteristics, such as gender, age, education, parental living status and religion. However, there were statistically significant differences in proportions between HIV-positive and HIV-negative youth on ever living on the streets (*χ*^2^ =10.14, df = 1, *p* = 0.002), past 12-month alcohol use (*χ*^2^ =16.38, df = 1, *p* < .0001), ever having sexual intercourse (*χ*^2^ =14.52, df = 1, *p* = 0.0001), ever engaging in sex work (*χ*^2^ =13.19, df = 1, *p* = 0.0003), inconsistent condom use in the past 3 months (*χ*^2^ =5.03, df = 1, *p* = 0.03), and ever being raped (*χ*^2^ =15.29, df = 1, *p* < 0.0001). A higher percentage of HIV-positive youth compared to HIV-negative youth reported ever living on the streets (33.6% vs. 20.7%, respectively), past 12-month alcohol use (47.4% vs. 29.1%, respectively), ever having sexual intercourse (69.8% vs. 51.2%, respectively), engaging in commercial sex work (12.4% vs. 2.2%, respectively), inconsistent condom use in the past 3 months (27.6% vs. 18.8%, respectively), and ever being raped (30.2% vs. 15.7%, respectively).

**Table 1:** Demographic characteristics and risk behaviors among self-reported HIV-positive and HIV-negative youth living in the slums of Kampala, *n* = 1103

	Self-reported HIV-positive (*n* = 116) 10.52%	Self-reported HIV-negative (*n* = 987) 89.48%	Chi-Square, *df*, *p*-value
Gender			
Male	48 (41.38%)	434 (43.97%)	.28, (*1*), *p* = .59
Female	68 (58.62%)	553 (56.03%)	
Age (IQR)	17 (3)	17 (3)	Z = 1.76, *p* = .08^a^
Education			
Never completed primary	30 (26.79%)	349 (35.69%)	3.55, (*2*), *p* = .17
Completed primary	31 (27.68%)	231 (23.62%)	
Secondary or higher	51 (45.54%)	398 (40.70%)	
Parental Living Status			
Both parents living	44 (37.93%)	402 (40.69%)	.35, (*2*), *p* = .84
One parent living	45 (38.79%)	371 (37.55%)	
Both parents dead	27 (23.28%)	215 (21.76%)	
Religion			
Christian-Catholic	34 (29.31%)	352 (35.63%)	2.11, (*3*), *p* = .55
Christian-Other	44 (37.93%)	322 (32.59%)	
Muslim	30 (25.86%)	249 (25.20%)	
Other religion	8 (6.90%)	65 (6.58%)	
Ever lived on the streets			
Yes	39 (33.62%)	204 (20.67%)	10.14, (*1*), *p* = .002
No	77 (66.38%)	783 (79.33%)	
Past 12-month alcohol use			
Yes	55 (47.41%)	287 (29.05%)	16.38, (*1*), *p* < .0001
No	61 (52.59%)	701 (70.95%)	
Ever had sexual intercourse			
Yes	81 (69.83%)	505 (51.17%)	14.52, (*1*), *p* = .0001
No	35 (30.17%)	482 (48.83%)	
Commercial sex work			
Yes	18 (22.22%)	62 (12.35%)	13.19, (*1*), *p* = .0003
No	98 (84.48%)	926 (93.72%)	
Condom use at last sex			
Yes	48 (41.38%)	340 (34.41%)	
No	68 (58.62%)	648 (65.59%)	2.21, (*1*), *p* = .14
Ever been raped			
Yes	35 (30.17%)	155 (15.69%)	15.29, (*1*), *p* < .0001
No	81 (69.83%)	833 (84.31%)	

Note. Thirty-one (*n* = 31) youth did not answer the self-reported HIV measure in the survey and were excluded from the analysis

^a^Wilcoxon two-sample test was used to compare ages between HIV-positive and HIV-negative youth

Table 2 presents alcohol use and alcohol-related behaviors among HIV-positive and HIV-negative youth. A higher proportion of HIV-positive youth were classified as problem drinkers, defined by the CAGE scores (21.6% vs. 13.9%, respectively). This difference was also statistically significant (*χ*^2^ =4.90, df = 1, *p* = 0.03). There were also statistically significant differences between HIV-positive and HIV-negative youth on age at first consumption of alcoholic beverage (*χ*^2^ =17.38, df = 4, *p* = 0.002) and age at first drunkenness (*χ*^2^ =27.28, df = 4, *p* < 0.0001). There were no statistically significant differences between HIV-positive and HIV-negative youth on frequency of alcohol beverages (*χ*^2^ =5.92, df = 3, *p* = 0.12); however, there were statistically significant differences with respect to the amount of alcohol consumed in a typical day (*χ*^2^ =38.60, df = 3, *p* < 0.0001). A higher percentage of HIV-positive youth reported consuming 3–4 drinks in a typical day when drinking compared to HIV-negative youth (28.4% vs. 9.3%, respectively).

**Table 2:** Alcohol use and alcohol-related behaviors among self-reported HIV-positive and HIV-negative youth living in the slums of Kampala, Uganda

	Self-reported HIV-positive	Self-reported HIV-negative	Chi-Square, *df*, *p*-value
Problem Drinker (defined by CAGE scores)			
Yes	25 (21.55%)	137 (13.87%)	4.90, (*1*), *p* = .03
No	91 (78.45%)	851 (86.13%)	
Age at consumption of first alcoholic beverage			
1–12	6 (5.22%)	52 (5.30%)	17.38, (*4*), *p* = .002
13–14	20 (17.39%)	94 (9.57%)	
15–16	25 (21.74%)	137 (13.95%)	
17–18	10 (8.70%)	56 (5.70%)	
Non-drinker	54 (46.96%)	643 (65.48%)	
Age at first drunkenness			
12	5 (4.31%)	18 (1.82%)	27.28, (*4*), *p* < .0001
13–14	14 (12.07%)	64 (6.48%)	
15–16	16 (13.79%)	108 (10.93%)	
17–18	17 (14.66%)	55 (5.57%)	
Never	64 (55.17%)	743 (75.20%)	
Frequency of alcoholic beverage consumption			
Monthly or less	5 (9.09%)	63 (21.95%)	5.92, (*3*), *p* = .12
2–4 times a month	19 (34.55%)	84 (29.27%)	
2–3 times a week	21 (38.18%)	107 (37.28%)	
4 or more times a week	10 (18.18%)	33 (11.50%)	
Frequency of full drinks consumed in typical day when drinking alcohol			
Non-drinker	54 (49.54%)	643 (69.21%)	38.60, (*3*), *p* < .0001
1–2 drinks	19 (17.43%)	174 (18.73%)	
3–4 drinks	31 (28.44%)	86 (9.26%)	
5 or more drinks	5 (4.59%)	26 (2.80%)	
Has a relative, friend, doctor, or other healthcare worker been concerned about your drinking or suggested you cut down?			
Yes	25 (46.30%)	91 (31.60%)	4.38, (*1*), *p* = .04
No	29 (53.70%)	197 (68.40%)	
Because of your own alcohol use, how often during the last month have you experienced any of the following?			
Had sex without a condom			
Never	24 (43.64%)	146 (50.69%)	16.70, (*2*), *p* = .0002
1–2 times	16 (29.09%)	118 (40.97%)	
3 or more times	15 (27.27%)	24 (8.33%)	
Had sex which you wished you hadn’t the next day			
Never	27 (49.09%)	151 (52.43%)	16.08, (*2*), *p* = .0003
1–2 times	12 (21.82%)	108 (37.50%)	
3 or more times	16 (29.09%)	29 (10.07%)	
Had sex with multiple partners			
Never	26 (47.27%)	200 (69.69%)	11.15, (*2*), *p* = .004
1–2 times	15 (27.27%)	52 (18.12%)	
3 or more times	14 (25.45%)	35 (12.20%)	
Ever been seriously injured or hurt due to your drinking?			
Yes	25 (46.30%)	91 (31.60%)	4.38, (*1*), *p* = .04
No	29 (53.70%)	197 (68.40%)	
Has someone else ever been seriously injured or hurt due to your drinking?			
Yes	20 (36.36%)	73 (25.44%)	2.78, (*1*), *p* = .10
No	35 (63.64%)	214 (74.56%)	

Table 2 also presents alcohol-related behaviors among self-reported HIV-positive and HIV-negative youth. The proportion of youth who reported having sex without a condom three or more times in the past 3 months, due to alcohol use, was significantly higher in the self-reported HIV-positive youth (27.3%) compared to HIV-negative youth (8.3%). Additionally, the proportion of youth who stated they had sex which they regretted the next day, due to alcohol use, was also higher among self-reported HIV-positive youth (29.1%) compared to HIV-negative youth (10.1%). The proportion of having sex with multiple partners three or more times in the past month, due to alcohol use, was higher among the self-reported HIV-positive youth (25.5%) compared to HIV-negative youth (12.2%). A higher proportion of self-reported HIV-positive youth compared to HIV-negative youth also reported injuries to themselves due to alcohol use (46.3% vs. 31.6%, respectively) and injuries to others due to alcohol use (36.4% vs. 25.4%, respectively).

Proportions in gender differences were computed among self-reported HIV-positive youth. Among HIV-positive youth, the majority were females (58.6%). The only statistically significant difference between HIV-positive males and females was reporting ever being raped (*χ*^2^ =15.17, df = 1, *p* < 0.0001). A higher percentage of females reported being raped compared to males (44.4% vs. 10.4%, respectively).

Table 3 presents the results of the bivariate and multivariable logistic regression analyses. In the bivariate analyses, ever living on the streets (OR: 1.93; 95% CI: 1.28, 2.94), commercial sex work (OR: 2.74; 95% CI: 1.56, 4.83), previously being raped (OR: 2.32; 95% CI: 1.51, 3.58), alcohol use (OR: 2.34; 95% CI: 1.46, 3.74), and problem drinking (OR: 2.10; 95% CI: 1.28, 3.47) were associated with self-reported HIV. In the multivariable analysis, previously being raped (OR: 1.70; 95% CI: 1.02, 2.83) and alcohol use without problem drinking (OR: 2.14; 95% CI: 1.24, 3.69).

**Table 3:** Demographic and psychosocial factors associated with self-reported HIV among youth living in the slums of Kampala, Uganda

	Unadjusted odds ratio (95% CI)	Adjusted odds ratio (95% CI)
Gender		
Male	1.00	1.00
Female	1.11 (0.75, 1.64)	1.03 (0.66, 1.59)
Age (IQR)	1.10 (0.98, 1.24)	1.03 (0.90, 1.19)
Education		
Never completed primary	1.00	1.00
Completed primary	1.56 (0.92, 2.65)	1.70 (0.98, 2.93)
Secondary or higher	1.49 (0.93, 2.39)	1.49 (0.89, 2.51)
Parental Living Status		
Both parents living	1.00	1.00
One parent living	1.11 (0.72, 1.72)	0.84 (0.52, 1.34)
Both parents dead	1.15 (0.69, 1.91)	1.49 (0.89, 2.51)
Religion		
Christian-Catholic	1.00	1.00
Christian-Other	1.42 (0.88, 2.27)	1.29 (0.79, 2.11)
Muslim	1.25 (0.74, 2.09)	1.49 (0.86, 2.58)
Other religion	1.27 (0.56, 2.88)	1.19 (0.49, 2.87)
Ever lived on the streets		
Yes	*1.93 (1.28, 2.94)*	1.55 (0.96, 2.49)
No	1.00	1.00
Commercial sex work		
Yes	2.74 (1.56, 4.83)	1.38 (0.68, 2.81)
No	1.00	1.00
Condom use at last sex		
Yes	1.35 (0.92, 1.99)	0.99 (0.63, 1.55)
No	1.00	1.00
Ever been raped		
Yes	*2.32 (1.51, 3.58)*	*1.70 (1.02, 2.83)*
No	1.00	1.00
Problem drinking		
Yes	*2.10 (1.28, 3.47)*	*1.76 (0.97, 3.19)*
No, but previously drank	*2.34 (1.46, 3.74)*	*2.14 (1.24, 3.69)*
Non-drinker	1.00	1.00

Note. Model fit statistics: *AIC* Akaike Information Criterion: 715.364, −2 Log Likelihood: 683.364, Wald Chi-Square: 38.21, *df* = 15, *p* = 0.0009

## Discussion

The purpose of this study was to determine the demographic characteristics, high-risk sexual behaviors, alcohol-related behaviors, and gender differences associated with self-reported HIV among youth living in the slums of Kampala, Uganda. Additionally, we sought to identify the associated factors with HIV among youth living in the slums of Kampala in the multivariable framework. The HIV prevalence among youth living in the slums of Kampala is higher than the national average (10.5% vs. 6.5%, respectively) [2]. Among youth living in the slums, the HIV epidemic is being fueled by alcohol use and alcohol-related sexual behaviors. Additionally, the prevalence of engaging in commercial sex work and previously being raped were much higher among HIV-positive youth compared to HIV-negative youth in our study. The organization which serves these youth, UYDEL, has many programs targeting HIV prevention and linkage to treatment. Additionally, UYDEL has vocational training programs, which help youth transition into sustainable jobs. Vocational training programs have often been implemented as HIV prevention initiatives to provide individuals with empowerment and economic activities, particularly among youth who may have previously resorted to commercial sex work, in order to prevent HIV transmission indirectly [34, 35].

Consistent with the literature, we found that the amount of alcohol consumed by youth was associated with HIV, and the frequency of alcohol was not associated with HIV [14]. Moreover, alcohol use in general was associated with HIV in the multivariable analysis whereas problem drinking was not statistically significantly associated with HIV. Alcohol has frequently been cited in the literature as a risk factor for the acquisition of HIV, through pathways involving inconsistent condom use and number of sexual partners [14, 20, 21]. The lack of significance between the amount of alcohol consumed with HIV suggests that any alcohol use, including consumption in smaller amounts, is associated with HIV. In our study, youth living with HIV reported a higher prevalence of inconsistent condom use attributable to alcohol use compared to HIV-negative youth. Future studies should examine the environmental contexts where youth consume alcohol during sexual activity in order to inform structural interventions to strengthen and enforce alcohol policies.

Our analyses did not find any statistical significant differences in proportions of demographic and psychosocial factors between male and female self-reported HIV-positive youth, with the exception of reporting previous rape. It is possible that due to the small sample of HIV-positive youth, we did not have the power to detect statistically significant differences. Moreover, it is also possible that these differences do not exist in this population. Young women and girls are disproportionately burdened by new HIV infections [1], and future studies should investigate if this disparity exists among youth living in the slums.

### Limitations

The convenience sample of youth is a key limitation of this study. However, these youth are often hard to reach, and a convenience sample may be one of the few strategies to sample these youth. Additionally, the youth in this study are seeking services at UYDEL, which may limit generalizability to other service-seeking youth living in the slums. Recall bias and social desirability bias may also impact misclassification of youth in this study, specifically for sensitive questions regarding sexual activity and alcohol use. HIV was also self-reported and may be underestimated in this sample, which is particularly concerning due to the high self-reported prevalence of HIV among the youth. While alcohol use was reported as highly prevalent among all youth in the sample, particularly among HIV-positive youth, this study did not assess the use of other substances. This may underestimate the impact of substance use on HIV acquisition among this population.

Due to the small sample of HIV-positive youth, gender differences among HIV-positive youth may not be accurately compared. Moreover, the small sample of HIV-positive youth may have limited our ability to detect statistically significant differences between HIV-positive and HIV-negative youth. Additionally, this study did not assess the mechanism which HIV was acquired in these youth (i.e., born with HIV vs. acquired via sexual transmission). Future studies should assess the mechanism of HIV acquisition, if known, in order to inform HIV prevention initiatives more comprehensively.

## Conclusions

Despite our noted limitations, this study is the first study to assess the broad context of HIV-positive youth living in the slums and streets of Kampala, Uganda. This study expands on the previous study regarding commercial sex work and HIV risk among youth living in the slums of Kampala [3], in addition to studies on alcohol use, rape, and violence among youth living in the slums of Kampala [3–7]. The extent of generalizability of this study to youth living in the slums and streets of other countries, or more broadly among youth in other countries, is not fully known. However, these youth report common characteristics that youth living in the slums of other countries also report, such as a lack of parental oversight, food scarcity, and limited infrastructure [4–7, 36, 37], which may enable generalizability to youth living in the slums of other countries in Africa.

These results will be used to inform interventions and policy planning in UYDEL and will aid in informing interventions for street and slum youth more broadly.

## Data Availability

The datasets generated and/or analyzed during the current study are not publicly available per institutional review board guidelines for data collection but are available from the corresponding author on reasonable request.

## Abbreviations

AIDS: Acquired Immunodeficiency Syndrome
AUDIT: Alcohol Use Disorders Identification Test (AUDIT)
HIV: Human immunodeficiency virus
Questionnaire CAGE: Questionnaire: a questionnaire consisting of 4 questions which comprise the acronym CAGE: (“Have you ever felt you should Cut down on your drinking?”; “Have people Annoyed you by criticizing your drinking?”; “Have you ever felt bad or Guilty about your drinking?”; and “Have you ever had a drink first thing in the morning to steady your nerves or to get rid of a hangover (Eye opener)?”)
UYDEL: Uganda Youth Development Link
